# The role of landscape evolution in the genetic diversification of a stream fish *Sarcocheilichthys parvus* from Southern China

**DOI:** 10.3389/fgene.2022.1075617

**Published:** 2023-01-06

**Authors:** Mingyue Li, Xishu Yang, Xiaomin Ni, Cuizhang Fu

**Affiliations:** Ministry of Education Key Laboratory for Biodiversity Science and Ecological Engineering, Coastal Ecosystems Research Station of the Yangtze River Estuary, Institute of Biodiversity Science and Institute of Eco-Chongming, School of Life Sciences, Fudan University, Shanghai, China

**Keywords:** phylogeography, genetic diversity, Southern China, stream capture, gobionidae, *sarcocheilichthys*

## Abstract

*Sarcocheilichthys parvus* (Cypriniformes: Gobionidae) is a stream fish which is endemic to sub-tropical coastal drainages in southern China, thus offering a valuable model for understanding how genetic divergence arises in stream-adapting freshwater fishes in this region. Using the mitochondrial Cyt *b* gene, integrative analyses of phylogeny, population demography, and ancestral area and paleo-drainage reconstructions are carried out to explicitly explore the role of landscape evolution in genetic diversification of *S. parvus*. The time-calibrated phylogeny of *S. parvus* indicates the splitting of two major lineages (A and B) at ∼3.66 Ma. Lineage A inhabits the Poyang Lake sub-drainage of the middle Yangtze River, Han River and Pearl River, and can be split into two sub-lineages (A-I and A-II), where sub-lineage A-II can be further sub-divided into three infra-sub-lineages (A-IIa, A-IIb and A-IIc). Except for the infra-sub-lineage A-IIc, which is restricted to the Han River and Pearl River, the other sub-lineages and infra-sub-lineages live exclusively in the Poyang Lake sub-drainage. Lineage B lives in the lower Yangtze River, Qiantang River, Jiaojiang River and Ou River, displaying close genetic relationships among the drainages. Rapid population expansion has occurred since the Late Pleistocene. Our findings indicate that the splitting of lineages A and B could be attributed to geographic isolation due to the Zhe–Min Uplift, acting as a biogeographic barrier before the late Early Pleistocene. Furthermore, the strong genetic divergence within Lineage A could be explained by the isolation role of the Nanling Mountains and Poyang Lake acting as an ecological barrier; while the lack of phylogenetic structure within Lineage B may have been the result of paleo-drainage connections or episodic freshwater connections during the eustatic low stand of sea level in the late Middle–Late Pleistocene.

## 1 Introduction

Phylogeographic research on freshwater fish has continued to play an important role in understanding the underlying causal factors shaping inter- and intra-specific genetic variations of freshwater fish biodiversity across contemporary isolated drainages (Shelley et al., 2020; Van Steenberge et al., 2020; Waters et al., 2020; Lima et al., 2021; Yang et al., 2022). Landscape evolution, either through tectonic activities or drainage rearrangements, has been considered to be a major driver for the diversification of obligate freshwater fishes (Burridge, et al., 2006; Unmack et al., 2013; Swartz et al., 2014; Xu et al., 2014; Zúñiga-Vega et al., 2014; Craw et al., 2016; Lima et al., 2017; Souza et al., 2020; Sholihah et al., 2021; Barreto et al., 2022; Souto-Santos et al., 2022; Val et al., 2022). One type of drainage rearrangement is stream capture, a geomorphological process that refers to a stream displacing a portion of another neighboring stream due to tectonic or erosive events (Bishop, 1995). Another type of drainage rearrangement is paleo-drainage connection, referring to river coalescence among contemporary coastal drainages during the eustatic low stand of sea level in the Pleistocene era (Burridge et al., 2008; Unmack et al., 2013; Thomaz et al., 2015). In addition, episodic freshwater connection may occur among neighboring coastal drainages through lowland flooding under short-term extreme weather events, especially on the wide continental shelf with low relief during the Pleistocene glacial period (Thacker et al., 2007; Burridge et al., 2008; Unmack et al., 2013; Zúñiga-Vega et al., 2014).

**FIGURE 1:**
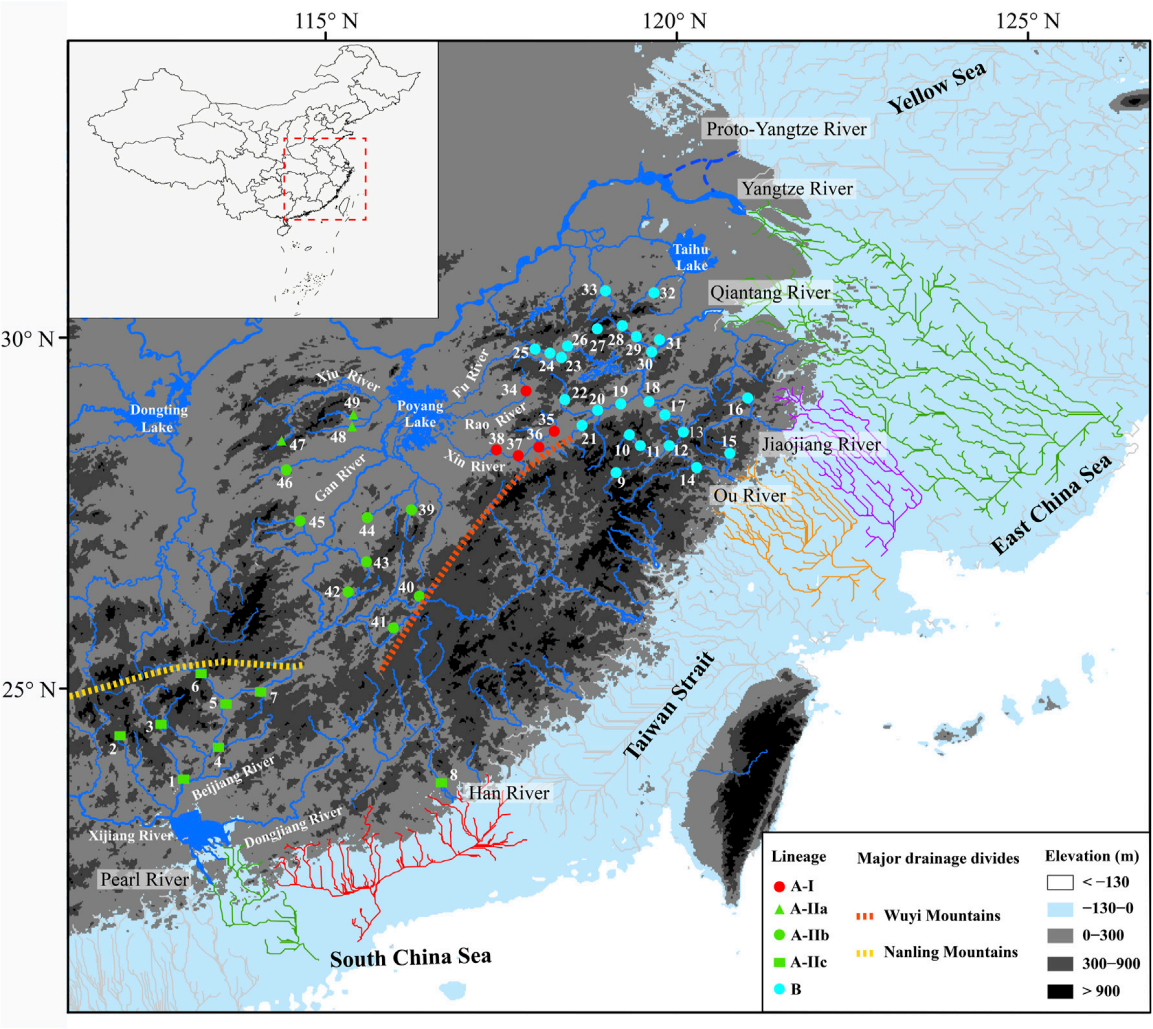
Map presenting 49 sampling localities of *S. parvus*. The distributions of lineages, sub-lineages, and infra-sub-lineages (defined in Figure 2) are shown with different colors or shapes. The area exposed during Last Glacial Maximum (largest height of sea level fall: 130 m) is shown in nattier blue, and the reconstructed paleo-drainages as distribution drainages for *S. parvus* in that area are presented by colored lines. Blue dotted lines indicate the possible routes of the proto-Yangtze River flowing into the Yellow Sea before the late Early Pleistocene (Zhang et al., 2019; Liu et al., 2022). Orange and yellow dotted lines indicate the major drainage divides.

The Yangtze River Basin is generally thought to lie in the northernmost part of southern China, based on delimitation of the northern boundary of the subtropical zone across China (Kou et al., 2020). After the uplift of the Nanling and Wuyi Mountains (Figure 1) around 15–10 Ma, the modern coastal drainages in southern China were established and have remained in a relatively stable configuration (Yan et al., 2018; Zhang et al., 2021). The northern species range limit for endemic freshwater fishes in southern China is often the Yangtze River (Chai and Fu, 2020; Yang et al., 2022). Stream capture, paleo-drainage connections, and episodic freshwater connections have been widely invoked to explain the genetic divergence, secondary contact, and range expansion of freshwater fishes among contemporary isolated drainages in southern China (Yang and He, 2008; Yang et al., 2009; Xu et al., 2014; Chen et al., 2017; Wang et al., 2021; Yang et al., 2022).

A long-term biogeographic barrier, the Zhe–Min Uplift (also called the Zhejiang–Fujian Uplift or Fukien–Reinan Massif), formed in the Late Mesozoic, and extends from the southern Korean Peninsula to Zhejiang and Fujian Provinces in southern China (Wageman et al., 1970; Jin and Yu, 1982). It was high terrain before the Pleistocene era and, thus, historically restricted the proto-Yangtze River (Figure 1) northward into the modern Yellow Sea (Chen and Stanley, 1995; Yi et al., 2014). Following the subsidence of the Zhe–Min Uplift after ∼0.8 Ma or ∼0.9 Ma, the proto-Yangtze River (Figure 1) began to divert from its northward flow, instead moving southward towards the East China Sea (Zhang et al., 2019; Liu et al., 2022). The role of the Zhe–Min Uplift has long been neglected in freshwater fish biogeographical studies regarding China. In this study, we first hypothesize that the existence or subsidence of the Zhe–Min Uplift before or after the late Early Pleistocene could impede or facilitate genetic exchange of freshwater fishes between the Yangtze River and its adjacent coastal drainages in southern China.

The Poyang Lake sub-drainage is located in the Poyang Lake Basin of the middle Yangtze River Basin, including the Poyang Lake and its surrounding five inflow rivers: The Fu, Rao, Xin, Gan and Xiu rivers (Figure 1). The Poyang Lake is the largest freshwater lake in China, with a water area of ∼4000 km^2^ during high water levels, and is connected with the Yangtze River by a narrow outlet (Shankman et al., 2006; Li et al., 2017). The Poyang Lake Basin formed in the Late Mesozoic, and its overall depression occurred in the Pleistocene era (Yang X. D. et al., 2016). The modern Poyang Lake may have emerged in the Holocene, but its precise age is still uncertain (Xu et al., 2019). Some authors have indicated that riverine impoundments (large reservoirs) create a gradient of hydrological and limnological conditions, functioning as ecological barriers against downstream movements for rheophilic freshwater fishes (Fluker et al., 2014; Pelicice et al., 2015). The results of a recent study indicated genetic differences in the freshwater gudgeon *Huigobio chenhsienensis* (Cypriniformes: Gobionidae) among the major tributaries flowing into the Poyang Lake (Yang et al., 2022). Therefore, we hypothesize that the Poyang Lake may serve as an ecological barrier, restraining dispersal and facilitating genetic divergence in stream-adapting freshwater fishes among the major tributaries within the Poyang Lake sub-drainage.

*Sarcocheilichthys parvus* (Cypriniformes: Gobionidae) is a small stream freshwater gudgeon with body length less than 7 cm (Supplementary Figure S1), which is endemic to sub-tropical coastal drainages in southern China (Yue, 1998; An et al., 2020). It has been known to be distributed south of the Yangtze River, including in the Poyang Lake sub-drainage of the middle Yangtze River, some tributaries of the lower Yangtze River, Qiantang River, Jiaojiang River, Ou River, Han River, and three sub-drainages (Xijiang, Beijiang and Dongjiang rivers) of the Pearl River (Li, 2018). *S. parvus* prefers sandy or pebbly substrate environments, swims on the sub-benthic water column, possesses an inferior mouth, and feeds on benthic invertebrate animals and algae (Xu, 2012). It is sexually mature at 2 years old, and spawns from March through August (Xu, 2012). Females of *S. parvus* possess a short ovipositor (Supplementary Figure S1), and likely lay their eggs through the inhalant siphon into the mantle cavity of unionid mussels, as has been observed in other *Sarcocheilichthys* species (Bǎnǎrescu and Nalbant, 1973; Barabanshchikov, 2004).

Despite much progress having been made in understanding how landscape evolution has shaped the phylogeographic patterns of freshwater fishes in southern China over time (Yang and He, 2008; Yang et al., 2009; Chen et al., 2017; Yang et al., 2022), a detailed picture of how their genetic divergence arises in this region is still poorly understood. For this study, we selected *Sarcocheilichthys parvus* as a model, in order to further improve our knowledge about the drivers and processes determining the intraspecific genetic variation of freshwater fishes in southern China. Using the mitochondrial cytochrome *b* (Cyt *b*) gene, integrative analyses of phylogeny, population demography, and ancestral drainage and paleo-drainage reconstructions were carried out to explicitly explore the role of landscape evolution in the genetic diversification of *S. parvus*. We also test special hypotheses regarding the Zhe–Min Uplift as a biogeographic barrier before the late Early Pleistocene and the Poyang Lake as an ecological barrier leading to genetic divergence of *S. parvus*.

## 2 Materials and methods

### 2.1 Specimen Collection

A total of 235 specimens from 49 localities covering the distributional range of *Sarcocheilichthys parvus* were collected, with the help of local fishermen, across the six coastal drainages in southern China from November 2011 to December 2019 (Figure 1; Supplementary Table S1). According to the laboratory animal—guideline for ethical review of animal welfare in China (GB/T 35892-2018), fish euthanasia was conducted using the anesthesia method. Sampled fish were anaesthetized with a 0.25 mL L^−1^ aqueous solution of Eugenol until they lost consciousness. Then, the unconscious fish were fixed in 75% ethanol. Subsequently, they were transferred into 95% ethyl alcohol for long-term storage, and deposited in the Zoological Museum of Fudan University.

### 2.2 Sequence acquirement

The total genomic DNA of *S. parvus* was extracted from muscle tissue of each specimen using a high-salt protocol (Miller et al., 1988). The mitochondrial genomes were amplified using 13 primer pairs (Supplementary Table S2), among which the 12th primer pair (GobND6F and GobProR) was used to amplify the Cyt *b* gene. Polymerase chain reactions (PCR) were carried out using 30.0 μL final volumes containing 15.0 μL 2 × Es Taq MasterMix, 0.6 μL of each primer with 20 μM, 1.2 μL genomic DNA and 12.6 μL dd H_2_O. All PCR reactions were run as follows: Thermal cycling began with 95°C denaturation for 5 min, followed by 35 cycles of 95°C denaturation for 50 s, 52.4°C–56.0°C annealing (Supplementary Table S2) for 60 s, 72°C extension for 70 s and, finally, 72°C extension for 10 min. The raw sequences were obtained by Sanger sequencing.

The sequences were assembled and trimmed using the Sequencher v5.4 software (Gene Codes, Ann Arbor, MI, USA), and manually adjusted if necessary. Nucleotide sequences for each gene were aligned using the MAFFT v7.427 software (Katoh and Standley, 2013). Haplotype calculations were performed with aligned sequences as input into the DnaSP v6.12.01 software (Rozas et al., 2017). The basic characteristics of aligned sequences were summarized using the MEGA v7.0.26 software (Kumar et al., 2016).

### 2.3 Phylogenetic inference and divergence time estimation

The time-calibrated phylogeny of *S. parvus* was inferred using haplotype sequences in the BEAST v2.6.6 software (Bouckaert et al., 2019). Due to a lack of fossil evidence for genus *Sarcocheilichthys*, a secondary calibration point was achieved through the mitogenome time tree of *S. parvus* and its relatives (Supplementary Figure S2), on the basis of two fossil species with intact skeletons and their corresponding stratigraphic time (mitogenome data sources provided in Supplementary Table S3). The bModelTest module was applied to automatically search for the best substitution model for each of 13 mitochondrial protein-coding genes (Bouckaert and Drummond, 2017). The first fossil species, †*Palaeogobio zhongyuanensi* (Zhou, 1990), occurring in the Eocene (50.5–42.0 Ma; Liu et al., 2018), corresponds to the ancestral node of Gobionidae and Acheilognathidae (i.e., calibration point 1; log-normal distribution, *μ* = 3.82 and *σ* = 0.05). Another fossil species, †*Gnathopogon macrocephala* (Zhou, 1990), occurring in the Miocene (20.4–15.0 Ma; Deng et al., 2019), corresponds to the ancestral node of *Gnathopogon* and *Coreoleuciscus* (i.e., calibration point 2; log-normal distribution, *μ* = 2.86 and *σ* = 0.078). The birth–death model and relaxed log-normal clock model were employed to specify the priors. Two duplicates of 400,000,000 MCMC generations were run, with a sample frequency of 10,000 and 30% burn-in. All parameters reached convergence (ESSs >200).

To infer the time-calibrated phylogeny of *S. parvus*, the divergence time between *S. parvus* and *Sarcocheilichthys caobangensis* (11.46 Ma with 95% confidence interval of 12.70–10.05 Ma; Supplementary Figure S2) was chosen as a secondary calibration point (normal distribution, *m* = 11.36 and *s* = 0.68). Based on the results of the molecular clock test (*X* ^2^ = 139.96, *df* = 98, *p* = 0.0035) accomplished by likelihood ratio test in the DAMBE v7.2.43 software (Xia and Xie, 2001), the relaxed log-normal clock model was used. *S. caobangensis* was used as the outgroup taxon. Two duplicates of 50,000,000 MCMC generations were run, with sample frequency of 1000 and 30% burn-in. The remaining settings were the same as above.

In addition, haplotype networks were obtained using the median-joining algorithm in the Network v10.2 software (Bandelt et al., 1999).

### 2.4 Genetic diversity and historical demography

Two genetic diversity indices, haplotype diversity (*h*) and nucleotide diversity (*π*), were calculated in the Arlequin v3.5.2.2 software (Excoffier and Lischer, 2010). The total genetic differentiation coefficient (*Φ*_ST_), pairwise *Φ*_ST_, and their significance among the drainages were obtained through 1000 random permutations and the Tamura–Nei model using Arlequin v3.5.2.2. Two genetic differentiation indices, N_ST_ and G_ST_, were obtained with 1000 random permutations in the PermutCpSSR v2.0 software (Pons and Petit, 1996). The population clusters with the largest genetic divergence were detected with setting of *K* = 2–48 in the SAMOVA v2.0 software (Dupanloup et al., 2002).

To reconstruct the historical demography of the lineage A and B, sequences of all individuals from each lineage were used. Bayesian skyline plots (BSP) were inferred in BEAST v2.6.6 and visualized in Tracer v1.7.0 (Rambaut et al., 2018), using the Coalescent Bayesian Skyline prior and a substitution rate for *Cyt b* (1.52% per site per million years, obtained from the time-calibrated tree of *S. parvus*). The generation time of *S. parvus* was set as 2 years (Xu, 2012). Therefore, the effective population size was calculated as follows: the relative value obtained by BSP analysis, multiplied by ten to the sixth power and divided by two. Two neutrality tests—Tajima’s *D* (Tajima, 1989) and Fu’s *Fs* (Fu, 1997)—were applied, and their significances were tested with 1000 random permutations. A mismatch distribution analysis was applied to detect demographic expansions through the evaluation of curve fitting and two parameters (i.e., roughness index and variance) in Arlequin v3.5.2.2 (Excoffier and Lischer, 2010).

### 2.5 Reconstructing ancestral area and paleo-drainages

To evaluate the biogeographic history of *S. parvus*, we reconstructed the ancestral area using the BioGeoBEARS v0.2.1 package (Matzke, 2013) in R v3.5.0. The time-calibrated phylogeny of *Cyt b* haplotypes was used as an input tree. Each drainage was defined as a discrete biogeographical area, and each haplotype were assigned based on sampling information (Supplementary Table S1). Six biogeographic models (DEC, DIVALIKE, BAYAREALIKE, and their derived models with +J parameter) were applied (Matzke, 2014), and the optimal model was identified by assessing the Akaike Information Criterion (AIC) (Akaike, 1973).

Due to the maximum sea level drop of about 130 m during the Last Glacial Maximum (Lambeck et al., 2014; Yokoyama et al., 2018), paleo-drainages related to the distribution drainages for *S. parvus* from the present coastline to the sea floor depth of −130 m were reconstructed using GIS technology, with reference to Thomaz et al. (2015). Using the ArcGIS v10.3 software, GEBCO_2014 Grid (30 arc-seconds interval) as input data were downloaded from the General bathymetric chart of the oceans (http://www.gebco.net/) project, and a series of hydrological tools were used to create visualized paleo-drainages [for further details, see Yang et al. (2022)].

## 3 Results

### 3.1 Phylogeny and divergence time

**FIGURE 2:**
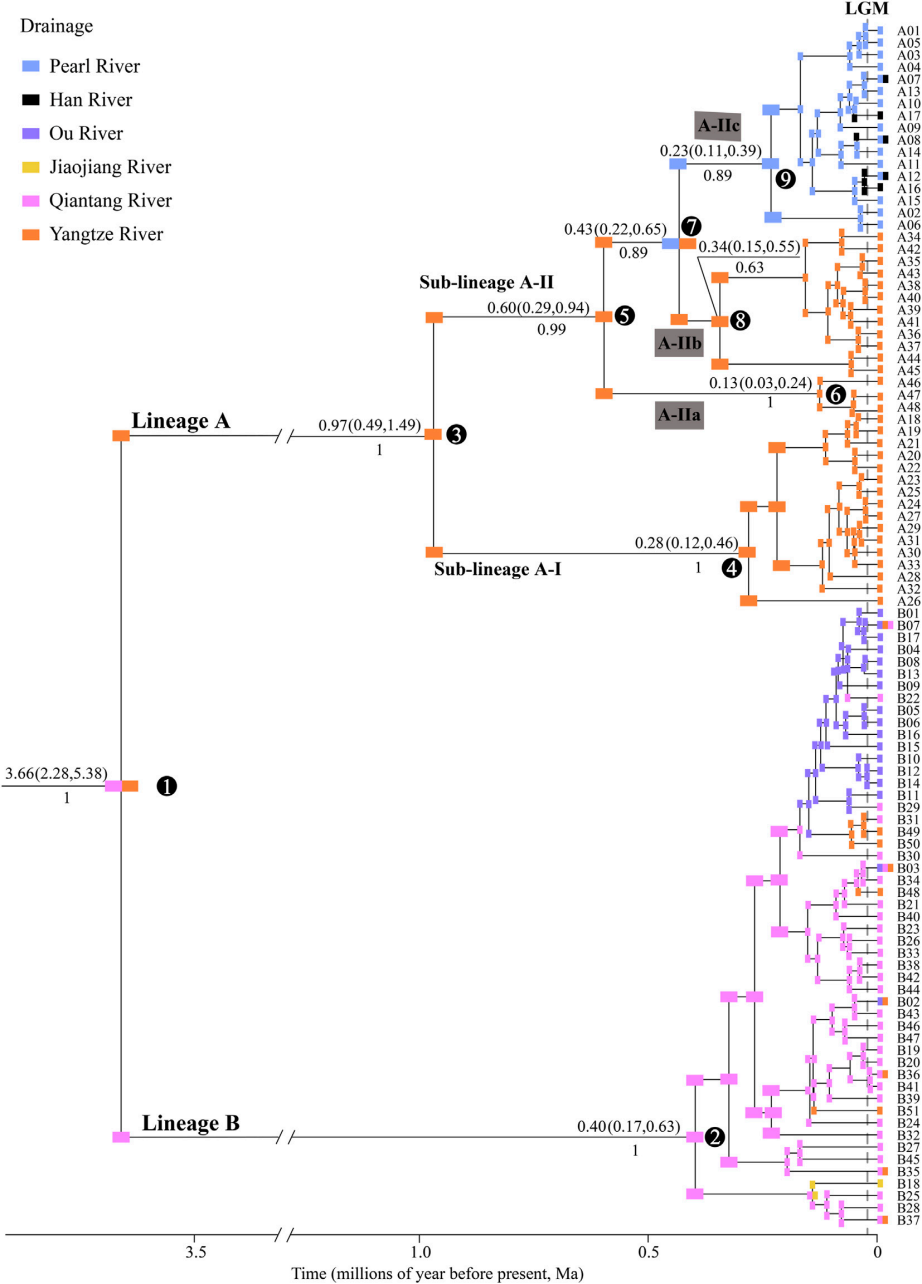
Time-calibrated Bayesian phylogeny of *S. parvus* and ancestral drainages of 99 *Cyt b* haplotypes. Ancestral drainages were inferred using the DIVALIKE model. Numbers above and below the nodes show the mean value and 95% confidence interval (CI) of divergence time and posterior probability, respectively. *S. caobangensis* was selected as the outgroup taxon (not shown). Numbers in circles represent the code for main nodes. LGM, Last Glacial Maximum.

A total of 99 haplotypes were identified in the *Cyt b* sequences (1140 bp; GeneBank no: ON964027–ON964125) from 235 individuals of *S. parvus* (Supplementary Table S1), which contained 157 variable sites and 123 parsimony informative sites. The time-calibrated phylogeny results displayed two major lineages (A and B), with a divergence time of 3.66 Ma (Figure 2). Lineage A included two sub-lineages (A-I and A-II), with a divergence time of 0.97 Ma, and sub-lineage A-I was further divided into three infra-sub-lineages (A-IIa, A-IIb and A-IIc) with divergence times of 0.60 and 0.43 Ma.

**FIGURE 3:**
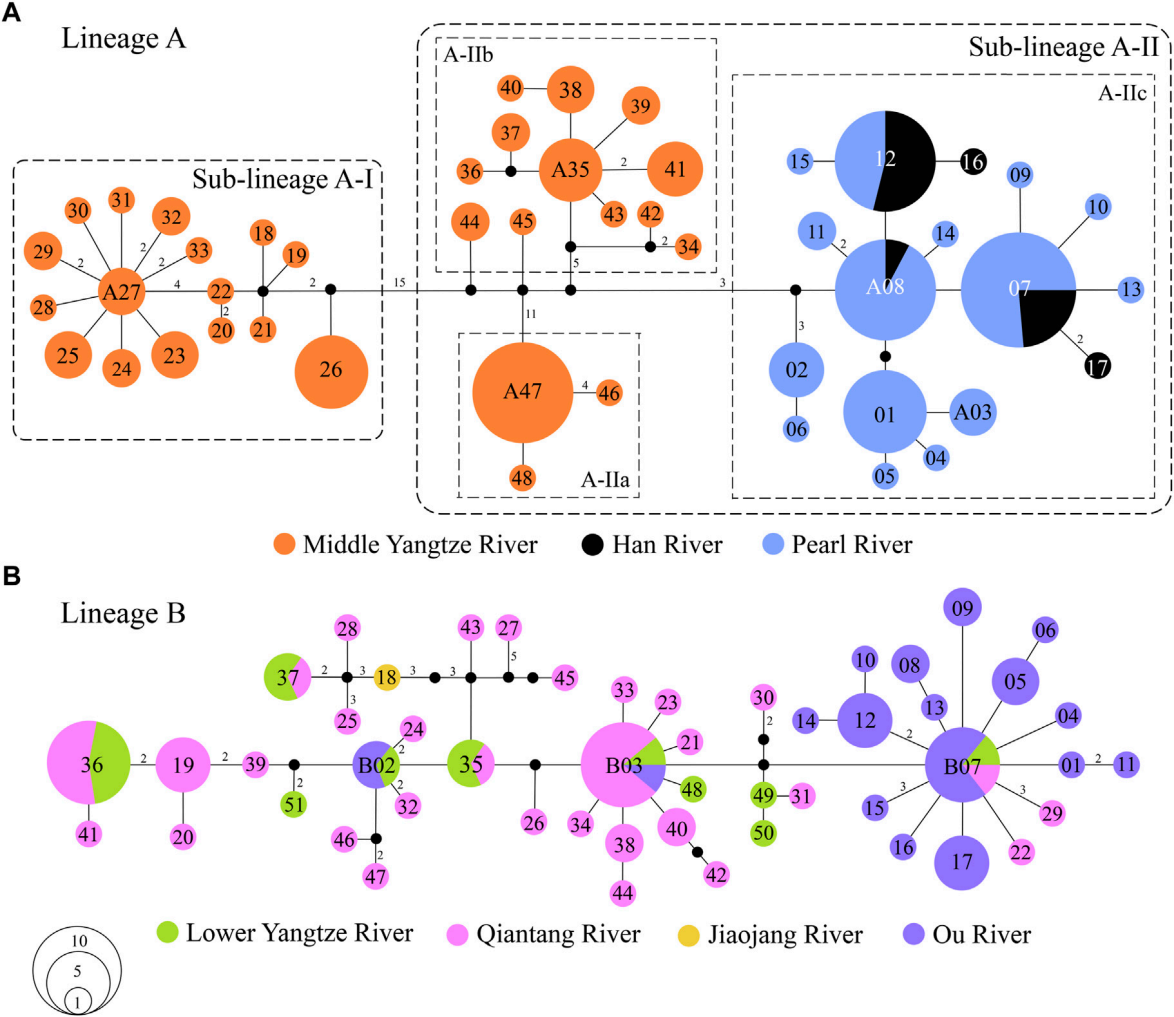
Median-joining networks of *S. parvus*: **(A)** Lineage A; and **(B)** Lineage B. The numbers in the circle represent the codes of haplotypes in the Lineage A and B. The mutation steps are shown as the numbers along the branches, except for one mutation step, and missing (unsampled) haplotype is presented by a black dot. The area of each circle is proportional to the number of individuals for each haplotype.

There were 62 mutation steps linking Lineage A with Lineage B (not shown in Figure 3). Lineage A inhabits the Poyang Lake sub-drainage of the middle Yangtze River, Pearl River and Han River (Figure 3A), while Lineage B is distributed in the lower Yangtze River, Qiantang River, Jiaojiang River, and Ou River (Figure 3B). Within Lineage A, sub-lineage A-I lives in the Rao and Xin rivers of the Poyang Lake sub-drainage (localities 34–38 in Figure 1); the infra-sub-lineages A-IIa and A-IIb are endemic to the Xiu and Gan rivers of the Poyang Lake sub-drainage, respectively (localities 47–49 and 39–46 in Figure 1); and infra-sub-lineage A-IIc inhabits the Pearl and Han rivers with three co-shared haplotypes (A07, A08 and A12; Figure 3A). Within Lineage B, six (B02, B03, B07 and B35–37) of the 51 haplotypes are located in two or three drainages, while the other haplotypes are restricted to a single drainage (Figure 3B).

### 3.2 Genetic diversity and population history

The haplotype diversity (*h*) ranged from 0.9623 (Qiantang River) to 0.7033 (Han River), while the nucleotide diversity (*π*) ranged from 0.0304 (Yangtze River) to 0.0013 (Han River) among the distribution drainages of *S. parvus* (Table 1).

**TABLE 1:** Genetic diversity for mitochondrial *Cyt b* of *S. parvus*.

Drainages	No. of samples	No. of haplotype	No. of private haplotype	Haplotype diversity	Nucleotide diversity
Pearl River	57	15	12	0.8716 ± 0.0225	0.0023 ± 0.0014
Han River	14	5	2	0.7033 ± 0.1008	0.0013 ± 0.0009
Ou River	32	17	14	0.9435 ± 0.0206	0.0024 ± 0.0014
Jiaojiang River	1	1	1	—	—
Qiantang River	46	31	26	0.9623 ± 0.0162	0.0049 ± 0.0026
Yangtze River	85	41	35	0.9605 ± 0.0109	0.0304 ± 0.0148
Overall	235	99	90	0.9783 ± 0.0031	0.0381 ± 0.0184

The total *Φ*_ST_ value was 0.714 (*p* = 0.000), and pairwise *Φ*_ST_ values among the drainages ranged from 0.105 (between Han River and Pearl River) to 0.970 (between Ou River and Han River; Table 2). N_ST_ and G_ST_ were 0.810 and 0.098, respectively. The amount of explained variation for the *Φ*_CT_ reached a plateau value (*Φ*_CT_ = 0.986) at *K* = 8 in the SAMOVA analysis. The best grouping arrangement corresponding to *K* = 8 was as follow. Group 1 (G1): Pearl River (localities 1–7; details for localities in Figure 1; Supplementary Table S1) + Han River (locality 8); Group 2 (G2): Ou River (localities 9–15); Group 3 (G3): Jiaojang River (locality 16) + Qiantang River (locality 17–31) + Lower Yangtze River (localities 32 and 33); Group 4 (G4): Rao River (locality 34) in the Poyang Lake sub-drainage of the Middle Yangtze River (PLMYR); Group 5 (G5): Xin River (localities 35–38) in the PLMYR; Group 6 (G6): localities 39–45 of Gan River in the PLMYR; Group 7 (G7): locality 46 of Gan River in the PLMYR; and Group 8 (G8): Xiu River (localities 47–49) in the PLMYR.

**TABLE 2:** Pairwise *Φ*_ST_ values (below diagonal) and the corresponding Bonferroni-corrected *p*-values (above diagonal) among the five drainages for *S. parvus*. The Jiaojiang River was excluded in this analysis, as only one specimen was collected in the drainage.

	Pearl river	Han river	Ou river	Qiantang river	Yangtze river
Pearl River		0.023	0.000	0.000	0.000
Han River	0.105		0.000	0.000	0.006
Ou River	0.966	0.970		0.000	0.000
Qiantang River	0.948	0.940	0.258		0.000
Yangtze River	0.314	0.242	0.653	0.651	

**FIGURE 4:**
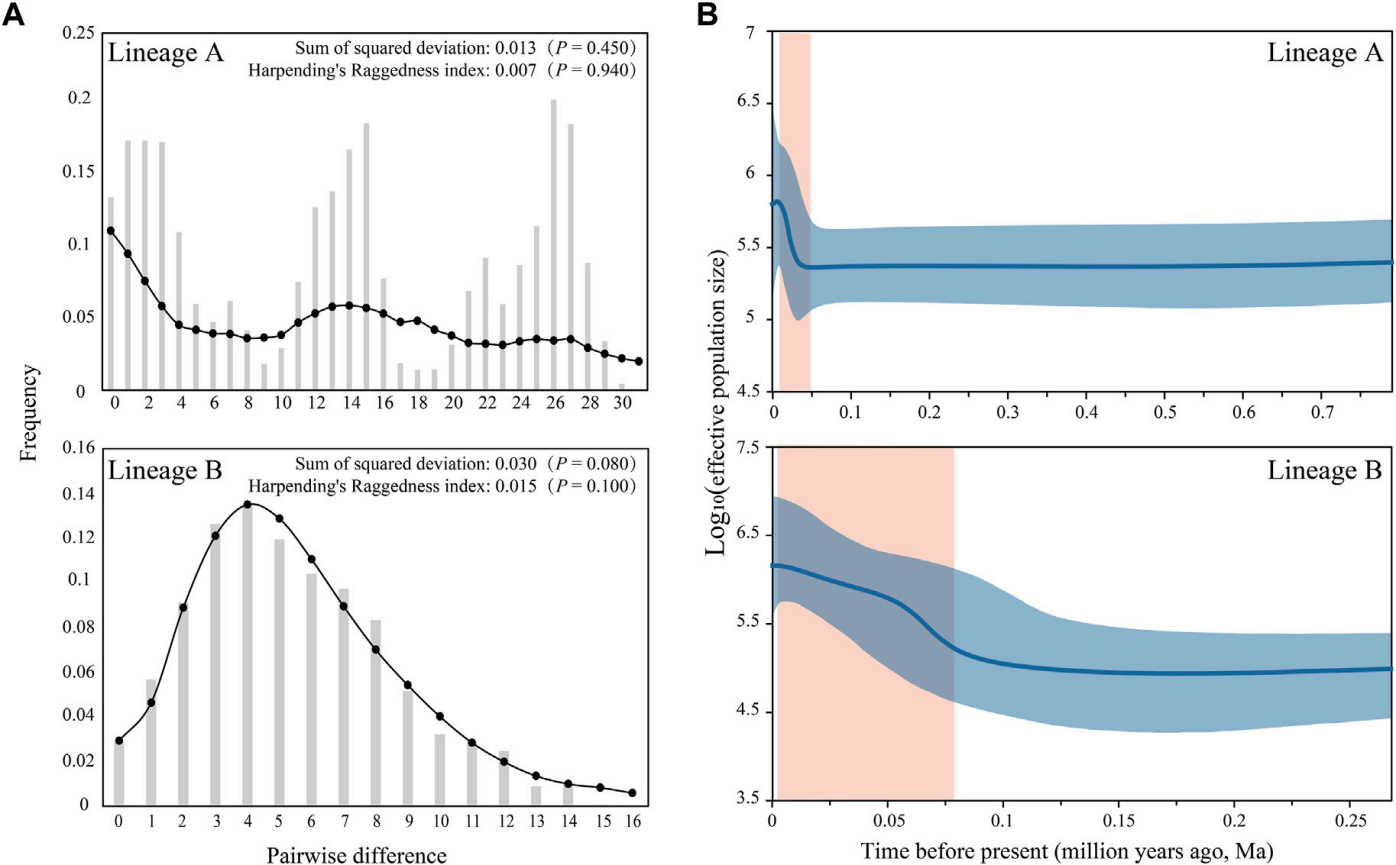
Population historic dynamics for lineages A and B of *S. parvus*: **(A)** Mismatch distribution; the bar and line with point represent the observed and predictive distributions, respectively. **(B)** Bayesian skyline plot; the blue line and shadow represent the median and 95% CI of effective population size, respectively, and the orange shadow represents the time frame of demographic expansion.

The estimated Tajima’s *D* and Fu’s *Fs* indices were –0.284 (*p* = 0.441) and –24.237 (*p* = 0.000) for Lineage A, and –1.978 (*p* = 0.006) and –25.262 (*p* = 0.000) for Lineage B. The mismatches exhibited multimodal and unimodal distributions for lineages A and B, respectively (Figure 4A). The BSP indicated that the population growth of lineages A and B began at 0.047 and 0.076 Ma, respectively (Figure 4B).

### 3.3 Ancestral area and paleo-drainages

Comparison of the biogeographical models demonstrated that the reconstruction results under the DIVALIKE model best explained the biogeographical history of *S. parvus*, based on the AIC value (Supplementary Table S4). *S. parvus* originated from the Qiantang and Yangtze rivers, and there were two major vicariant events on nodes 1 and 7, as well as multiple dispersal events within nodes 2 and 9 (Figure 2).

The results of coastal paleo-drainage reconstruction on the exposed continental shelf during the Last Glacial Maximum indicated that the Qiantang and Yangtze rivers were interconnected, whereas the Pearl, Han, Ou and Jiaojiang rivers independently entered into the sea, considering the distribution drainages of *S. parvus*.

## 4 Discussion

### 4.1 Major drivers of the lineage diversification

In this study, *S. parvus* from each of the six isolated drainages was found not to be a monophyletic group. Instead, our analyses revealed two major lineages—A and B—with deep genetic divergence, where the distributions of the two lineages exhibited reciprocal separations over geographic space (Figure 1). Lineages A and B originated in the Yangtze River and the Qiantang River, respectively. The phylogeographic pattern in *S. parvus*—namely*,* “deep gene tree, major lineages allopatric” (Avise, 2000)—could be explained by the existence of a long-term biogeographic barrier, the Zhe–Min Uplift (for details, see the Introduction), preventing genetic exchange between the Yangtze and Qiantang rivers. Our timing results regarding the divergence between the two major lineages A and B in *S. parvus* during the late Early Pliocene pre-dated the subsidence of the Zhe–Min Uplift in the late Early Pleistocene (Shankman et al., 2006; Zhang et al., 2019). Therefore, the long-term isolation between the Yangtze River and Qiantang River could have led to the deep genetic divergence between the two major lineages A and B in *S. parvus*. Our findings indicate that the presence of the Zhe–Min Uplift before the late Early Pleistocene likely played an important role in the genetic divergence of freshwater fishes between the Yangtze River and its adjacent coastal drainages in southern China.

In Lineage A, *S. parvus* exhibited a strong phylogenetic structure among the major tributaries (i.e., the Rao, Xin, Gan and Xiu rivers) flowing into the Poyang Lake, in the apparent absence of physical barriers (Figure 1). Our timing results for the divergence between sub-lineages A-I (the Rao and Xin rivers) and A-II (the Gan and Xiu rivers) at ∼0.97 Ma (node 2 in Figure 2), and between the infra-sub-lineages A-IIa (the Xiu River) and A-IIb (the Gan River) at ∼0.60 Ma (node 5 in Figure 2), was in accordance with warm and humid climates over the period ca. 1.2–0.9 Ma (Chen D. B. et al., 2020) and ca. 0.7–0.35 Ma in southern China (Hong et al., 2013; Ao et al., 2020). Therefore, the large Poyang Lake, with high water levels, may have formed due to warm and humid climates during the late Early and early Middle Pleistocene (Xu et al., 2019). This large lake may have then become an ecological barrier, impeding the dispersal of and facilitating the genetic divergence of *S. parvus* among the major tributaries within the Poyang Lake sub-drainage. More studies on genetic structure of fishes and other animals are needed to make generalization on the role of Poyang Lake acted as a barrier for driving intraspecific divergence. In addition, our findings may also provide empirical evidence with respect to natural ecosystems, supporting a prior theory that large reservoirs create a gradient of hydrological and limnological conditions to function as ecological barriers against the downstream movements of rheophilic fishes (Pelicice et al., 2015).

Lineage A of *S. parvus* originated in the Yangtze River, subsequently moving into the Pearl and Han rivers to form the infra-sub-lineage A-IIc; in particular, this infra-sub-lineage originated in the Pearl River and, subsequently, moved into the Han River, based on the ancient area reconstruction results (Figure 2). Our timing results for the divergence between infra-sub-lineages A-IIb and A-IIc was at ∼0.43 Ma (node 7 in Figure 2), when the global sea level was ∼100 m lower than the present day (Grant et al., 2014), and it suggested no reciprocal connections among the Yangtze, Han and Pearl rivers based on paleo-drainage reconstructions during the Last Glacial Maximum (LGM, Figure 1). Therefore, our findings indicate that a headwater stream section belonging to the Gan River of the Yangtze River drainage was likely captured by the Beijiang River of the Pearl River drainage (Figure 1) in the Middle Pleistocene, although there is a lack of geological evidence, and the episodic river capture event could be responsible for the range expansion of *S. parvus* across the Nanling Mountains (Figure 1). And then, the isolation role of the Nanling Mountains could result in genetic divergence between the infra-sub-lineage A-IIb living in the Gan River of the Yangtze River drainage and A-IIc inhabiting the Pearl and Han rivers. Stream capture across drainage divides has also been widely invoked to explain range expansion of freshwater fishes through vicariance processes (Burridge et al., 2006; Yang et al., 2009; Xu et al., 2014; Kim et al., 2017; Lima et al., 2017; Souza et al., 2020; Lima et al., 2021; Barreto et al., 2022). The close genetic relationships between the Pearl River and Han River in *S. parvus* has also been observed in another freshwater gudgeon, *Squalidus argentatus* (Yang et al., 2012). Although the lack of coalescence between the Pearl and Han rivers during the historically low sea level period in the LGM (Figure 1), the close genetic relationships between the two neighboring drainages could result from episodic freshwater connections under extreme weather events—for example, through lowland flooding—as has been suggested in other fish studies in southern China (Yang J. Q. et al., 2016; Yu et al., 2016; Chen W. T. et al., 2020).

Lineage B of *S. parvus* originated in the Qiantang River, then dispersed into the lower Yangtze River, Jiaojiang River, and Ou River, based on the ancient area reconstruction results (Figure 2). The geographic distributions of Lineage B display a few haplotypes across two or three drainages, while other haplotypes are confined to a single drainage (Figure 3B). Our timing of the crown age of the Lineage B at ∼0.40 Ma (node 2 in Figure 2) indicates that the phylogeographic pattern in Lineage B could be explained by recent historical connections among the lower Yangtze River, Qiantang River, Jiaojiang River, and Ou River (Chen et al., 2017; Ding et al., 2020; Yang et al., 2022). The recent historical connections between the Yangtze and Qiantang rivers are supported by the coalescence of the two rivers during the Last Glacial Maximum (Figure 1). However, the paleo-drainage reconstruction results indicated no coalescence among the Qiantang, Jiaojiang, and Ou rivers during the historically low sea level period (Figure 1); therefore, the close genetic relationships may have resulted from episodic sheet flow or episodic tributary connections among the three isolated neighboring rivers under short-term weather events, and that these episodes may have occurred on the wide continental shelf with low relief during the glacial period of the Late Middle–Late Pleistocene, as has been suggested in previous studies (Thacker et al., 2007; Burridge et al., 2008; Unmack et al., 2013; Zúñiga-Vega et al., 2014). A similar scenario has also been revealed by a recent study, in which the close genetic relationships in another freshwater gudgeon *Huigobio chenhsienensis* across the lower Yangtze River, Jiaojiang River, and Ou River have been suggested to be a result of lowland flooding during the eustatic low stand of sea level in the late Middle Pleistocene (Yang et al., 2022). In addition, our timing of the crown age of the Lineage B in *S. parvus* during the Middle Pleistocene post-dated the southward migration of the proto-Yangtze River into East China Sea, forming the modern Yangtze estuary in the late Early Pleistocene due to the subsidence of the Zhe–Min Uplift (Zhang et al., 2019; Liu et al., 2022). Therefore, our findings indicate that the subsidence of Zhe–Min Uplift after the late Early Pleistocene facilitated gene flow in freshwater fishes between the Yangtze River and its adjacent coastal drainages in southern China.

### 4.2 Genetic diversity and population history

The N_ST_ was much larger than the G_ST_ in *S. parvus*, suggesting a clear phylogeographic signature. Grant and Bowen (Grant and Bowen, 1998) have suggested that geographic co-occurrence between previously allopatric lineages could result in large values of nucleotide diversity. In our case, the Yangtze River had higher nucleotide diversity due to geographic co-occurrence of the two major lineages A and B in *S. parvus* (see Section 4.1), while other drainages characterized by a single lineage (A or B), such as the Pearl, Han, Ou, or Qiantang rivers, displayed lower nucleotide diversity. The high pairwise *Φ*_ST_ observed among the drainages in *S. parvus* were in line with that observed for other freshwater fishes from southern China (Chen et al., 2007; Yang and He, 2008; Watanabe et al., 2010; Wu et al., 2013; Yu et al., 2016; Yang et al., 2022).

The signature of recent population expansion was evident in the negative Tajima’s *D* and Fu’s *Fs* indices, as well as the unimodal mismatch distributions in *S. parvus*. Meanwhile, the respective population growth of the lineages A and B began at approximately ∼0.047 and ∼0.076 Ma (Figure 4). Paleoclimate studies demonstrated that the period between 0.076 and 0.047 Ma in the Late Pleistocene was in an interglacial to glacial transition stage, when the climate was cold and dry, and the sea level dropped down ∼20–65 m in comparison with the present day (Grant et al., 2014; Cheng et al., 2016). Therefore, episodic sheet flow through lowland flooding or episodic tributary connections among the isolated neighboring rivers under short-term weather events in the Late Pleistocene could result in spatial expansion to facilitate rapid population growth of lineages A and B. The timing difference of population expansion for lineages A and B may reflect the different biogeographical process. The pattern of rapid population expansion in the Late Pleistocene prior to the Last Glacial Maximum has been commonly reported in previous studies on freshwater fishes in East Asia (Yang et al., 2009; Watanabe et al., 2010; Xu et al., 2014; Chen et al., 2017; Zheng and Yang, 2018; Yang et al., 2022).

### 4.3 Implications for conservation

The protection of genetic diversity and maintaining evolutionary processes across the ranges of species have been recognized as critical components for biodiversity conservation in the face of global changing environments (Coates et al., 2018; Laikre et al., 2020). The results of our SAMOVA analysis indicated that eight conservation units (G1–G8) should be considered for the conservation of *S. parvus*. However, the phylogeographic breaks with long-term separations in the *Cyt b* gene tree of *S. parvus* (Figure 2) indicate that the two major lineages (A and B), two sub-lineages (A-I and A-II), and three infra-sub-lineages (A-IIa, A-IIb, and A-IIc) could be recognized as evolutionary significant units (ESUs) or management units (MUs) (Moritz, 1994; Avise, 2005). Our identified multiple ESUs or MUs and five conservation units (G4–G8) are located in different tributaries flowing into the Poyang Lake sub-drainage of the middle Yangtze River. Therefore, our results highlight that the Poyang Lake sub-drainage should be considered as area of spatial conservation prioritization for the protection of the genetic diversity of *S. parvus* (Andrello et al., 2022). Our findings also indicated the importance of considering the spatial complexity of the large River drainages when developing management and conservation strategies for maintaining the genetic diversity of freshwater fishes in southern China, as has been suggested by a recent study emphasizing the effects of ecosystem size and spatial complexity in co-regulating riverine biodiversity in nature (Terui et al., 2021).

## 5 Conclusion

In summary, *S. parvus* was found to be comprised of two major lineages (A and B), displaying strong phylogeographic structure. The splitting of lineages A and B was attributed to geographic isolation, due to the Zhe–Min Uplift acting as a biogeographical barrier before the late Early Pleistocene. Within lineage A, the strong genetic divergence in the Poyang Lake sub-drainage of the middle Yangtze River could be explained by Poyang Lake acting as an ecological barrier, and historical river capture was the main driver of the range expansion of *S. parvus* from the Yangtze River into the Pearl and Han rivers. Within lineage B, the lack of phylogenetic structure among the lower Yangtze River, Qiantang River, Jiaojiang River, and Ou River may have resulted from paleo-drainage connections or episodic freshwater connections during the eustatic low stand of sea level in the Late Middle–Late Pleistocene. Our results also provide new insight into the planning of management and conservation strategies for preserving the genetic diversity of freshwater fishes in southern China. More phylogeographic studies are needed to draw generalizations on the role of the Zhe–Min Uplift as a biogeographic barrier and Poyang Lake as an ecological barrier in the biogeographic processes of freshwater fishes in southern China.

## Data Availability

The data presented in the study are deposited in the GenBank repository of the national center for biotechnology information (NCBI), accession number ON964027-ON964125, ON963980.
