# Supplementary material for: The role of landscape evolution in the genetic diversification of a stream fish Sarcocheilichthys parvus from Southern China
Source: Front Genet. 2023 Jan 6;13:1075617. doi: 10.3389/fgene.2022.1075617 (PMC9853433; doi:10.3389/fgene.2022.1075617)
Supplement: Supplementary file 1 [file DataSheet1.pdf]

Supplementary Material

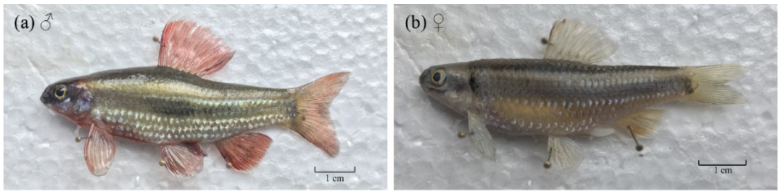

Supplementary Figure S1. *Sarcocheilichthys parvus*. (a) Male and (b) Female. The two specimens from the Jixi County, Anhui Province. Photographs by Mingyue Li on June 12, 2019.

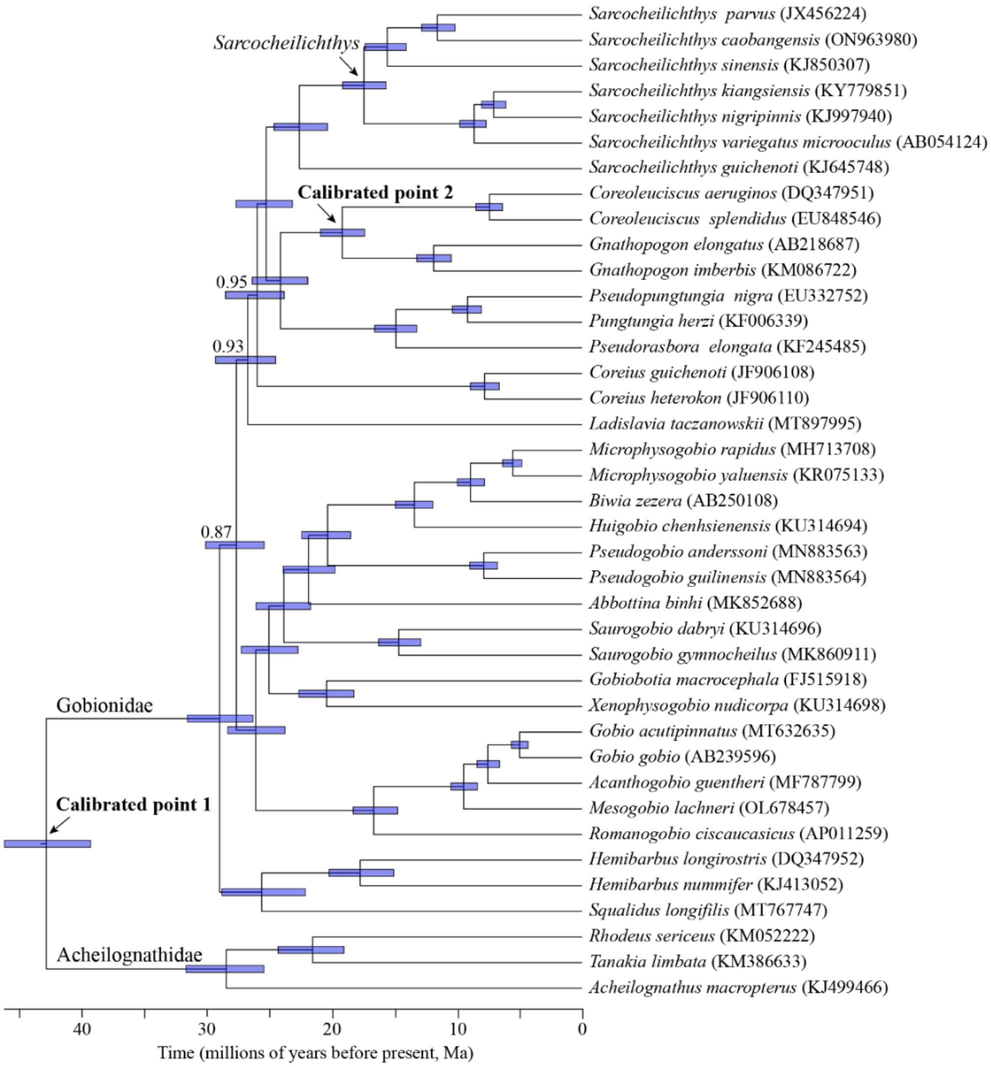

Supplementary Figure S2. Time-calibrated Bayesian phylogeny of the genus *Sarcocheilichthys* and its relatives. The posterior probability equal to 1 was not shown, and other values shown above the branch. The bars on the nodes indicate the 95% confidence interval of divergence times.

Supplementary Table S1. Information on specimens of *S. parvus* and Cyt *b* haplotypes.

Locality	Latitude	Longitude	n	*Cyt b* haplotype
Pearl River				
1. Qingyuan City, Guangdong Province	23.69 °N	113.03 °E	3	A01(2), A02(1)
2. Lianshan County, Guangdong Province	24.28 °N	112.02 °E	9	A01(4), A02(1), A03(2), A04(1), A05(1)
3. Yangshan County, Guangdong Province	24.47 °N	112.67 °E	7	A01(3), A02(2), A03(1), A06(1)
4. Yingde City, Guangdong Province	24.18 °N	113.42 °E	15	A07(7), A08(4), A09(1), A10(1), A11(1), A12(1)
5. Shaoguan City, Guangdong Province	24.81 °N	113.59 °E	17	A07(4), A08(8), A11(1), A12(3), A13(1)
6. Lechang City, Guangdong Province	25.29 °N	113.04 °E	5	A07(1), A12(2), A14(1), A15(1)
7. Shixing County, Guangdong Province	24.93 °N	114.11 °E	1	A07(1)
Han River				
8. Chaozhou City, Guangdong Province	23.68 °N	116.63 °E	14	A07(4), A08(1), A12(7), A16(1), A17(1)
Ou River				
9. Longquan City, Zhejiang Province	28.08 °N	119.13 °E	4	B01(1), B02(2), B03(1)
10. Suichang County, Zhejiang Province	28.62 °N	119.32 °E	4	B04(1), B05(1), B06(1), B07(1)
11. Songyang County, Zhejiang Province	28.46 °N	119.48 °E	6	B07(2), B08(2), B09(1), B10(1)
12. Lishui City, Zhejiang Province	28.46 °N	119.89 °E	6	B05(1), B07(1), B11(1), B12(2), B13(1)
13. Jinyun County, Zhejiang Province	28.66 °N	120.09 °E	6	B05(1), B09(1), B12(1), B14(1), B15(1), B16(1)
14. Qingtian County, Zhejiang Province	28.15 °N	120.28 °E	2	B07(1), B12(1)
15. Yongjia County, Zhejiang Province	28.35 °N	120.75 °E	4	B17(4)
Jiaojang River				
16. Tiantai County, Zhejiang Province	29.14 °N	121.03 °E	1	B18(1)
Qiantang River				
17. Wuyi County, Zhejiang Province	28.90 °N	119.83 °E	3	B03(1), B19(1), B20(1)
18. Jinhua City, Zhejiang Province	29.09 °N	119.60 °E	4	B21(1), B22(1), B23(1), B24(1)
19. Longyou County, Zhejiang Province	29.06 °N	119.20 °E	2	B03(1), B25(1)
20. Quzhou City, Zhejiang Province	28.97 °N	118.87 °E	2	B03(1), B26(1)
21. Jiangshan City, Zhejiang Province	28.75 °N	118.65 °E	2	B27(1), B28(1)
22. Kaihua County, Zhejiang Province	29.12 °N	118.10 °E	3	B29(1), B30(1), B31(1)
23. Huangshan City, Anhui Province	29.72 °N	118.31 °E	1	B32(1)
24. Xiuning County, Anhui Province	29.78 °N	118.19 °E	1	B33(1)
25. Yixian County, Anhui Province	29.84 °N	117.98 °E	2	B03(1), B19(1)
26. Shexian County, Anhui Province	29.88 °N	118.44 °E	3	B03(2), B34(1)
27. Jixi County, Anhui Province	30.06 °N	118.60 °E	11	B35(1), B36(5), B37(1), B38(1), B39(1), B40(1), B41(1)
28. Lin'an City, Zhejiang Province (Changhua Town)	30.17 °N	119.22 °E	4	B07(1), B19(1), B40(1), B42(1)
29. Lin'an City, Zhejiang Province (Yuqian Town)	30.19 °N	119.40 °E	2	B38(2), B42(1)
30. Tonglu County, Zhejiang Province	29.80 °N	119.64 °E	3	B03(2), B44(1), B45(1)
31. Hangzhou City, Zhejiang Province (Xindeng Town)	29.97 °N	119.75 °E	3	B19(2), B46(1), B47(1)
Lower Yangtze River				
32. Anji County, Zhejiang Province	30.64 °N	119.67 °E	1	B02(1)
33. Ningguo City, Anhui Province	30.67 °N	118.98 °E	14	B03(1), B07(1), B35(2), B36(4), B37(2), B48(1), B49(1), B50(1), B51(1)
Middle Yangtze River				
34. Wuyuan County, Jiangxi Province (Rao River)	29.24 °N	117.85°E	5	A18(4), A19(1), A20(1), A21(1), A22(1)
35. Yushan County, Jiangxi Province (Xin river)	28.67 °N	118.25 °E	9	A23(2), A24(1), A25(1), A26(3), A27(2)
36. Shangrao City, Jiangxi Province (Xin river)	28.44 °N	118.18 °E	8	A23(1), A25(1), A26(2), A28(1), A29(1), A30(1), A31(1)

37. Yanshan County, Jiangxi Province (Xin river)	28.32 °N	117.74 °E	8	A24(1), A25(1), A26(2), A29(1), A32(2), A33(1)
38. Yiyang County, Jiangxi Province (Xin river)	28.40 °N	117.43 °E	1	A27(1)
39. Yihuang County, Jiangxi Province (Gan River)	27.55 °N	116.22 °E	1	A34(1)
40. Shicheng County, Jiangxi Province (Gan River)	26.32 °N	116.33 °E	2	A35(2)
41. Ruijin City, Jiangxi Province (Gan River)	25.87 °N	116.03 °E	4	A36(1), A37(1), A38(1), A39(1)
42. Xingguo County, Jiangxi Province (Gan River)	26.38 °N	115.32 °E	4	A35(3), A40(1)
43. Yongfeng County, Jiangxi Province (Gan River)	26.81 °N	115.58 °E	6	A37(1), A39(1), A41(4)
44. Chongren County, Jiangxi Province (Gan River)	27.44 °N	115.59 °E	1	A42(1)
45. Anfu County, Jiangxi Province (Gan River)	27.39 °N	114.63 °E	3	A38(2), A43(1)
46. Wanzai County, Jiangxi Province (Gan River)	28.12 °N	114.44 °E	3	A44(2), A45(1)
47. Tonggu County, Jiangxi Province (Xiu River)	28.53 °N	114.39 °E	1	A46(1)
48. Fengxin County, Jiangxi Province (Xiu River)	28.71 °N	115.38 °E	2	A47(2)
49. Jing'an County, Jiangxi Province (Xiu River)	28.85 °N	115.39 °E	12	A47(11), A48(1)

Supplementary Table S2. Thirteen primer pairs used for the amplification of mitochondrial genes.

Primer pair	Forward primer	Reverse primer	Annealing temperature	Source
1	Gob12SF: 5'-AAGGCATGGTCCYGACCTTA-3'	Gob16sR: 5'-TTCGGTAGGTCTRTCACTTC-3'	55.4 ºC	(Chai et al., 2020)
2	GobValF: 5'-ACACCGAGAAGACATCCA-3'	GobLeuR1: 5'-GGGAAGAGGAYTTGAACC-3'	54.3 ºC	this study; (Chai et al., 2020)
3	GobND1F: 5'-GCAGCCGCTATTAAGGGTT-3'	GobND1R1: 5'-GGRTTCATTGATGGAGGA-3'	53.3 ºC	(Chai et al., 2020)
4	GobIleF: 5'-GCCCAAGGACCACTTTGATAG-3'	GobCOIR: 5'-CCAAATACRAGATARAGGGT-3'	52.5 ºC	(Chai et al., 2020)
5	AsnF: 5'-AGCGAGCATCCATCTACTT-3'	ARSerR: 5'-GGTYATGTGACTGGCTTGA-3'	54.6 ºC	(Chai et al., 2020)
6	GobCOIF: 5'-TGAGAAGCCTTYGCCGCYAAACG-3'	TGobATP6R: 5'-GCTTGGTGTGCCATTARACGTTTTCTTG-3'	52.4 ºC	(Chai et al., 2020; Yang et al., 2022)
7	SaATP6F: 5'-GAAACAATTAGCCTCTT-3'	GobArgR: 5'-CTGAGYCGAAATCAGAGG-3'	52.5 ºC	this study; (Chai et al., 2020)
8	SaGlyF: 5'-CTTCCAATYATTTAGYCTTGG-3'	SaND4R: 5'-ATAGGCTGTGGGCRGTTGTTGC-3'	53.5 ºC	this study
9	GobND4F: 5'-TAGCATTTCAYCGCACMC-3'	GobLeuR: 5'-TGGAYTTGCACCAAGAGT-3'	54.5 ºC	(Chai et al., 2020)
10	GobSerF1: 5'- ACTYACCRAGGAAGGACA-3'	GobND5R: 5'-TCCYCAGGCAAGYCGTTT-3'	54.0 ºC	(Chai et al., 2020)
11	SaND5F: 5'-CTAAACACCTCCYACCT-3'	SaCytbR: 5'-AAGAATGATGCTCCGTT -3'	52.8 ºC	this study
12	GobND6F: 5'-AAAATAGGTCATAATTCTTGCTCGG-3'	GobProR: 5'-GTTTAATTTAGAATTCTGGCTTTGG-3'	56.0 ºC	(Chai et al., 2020)
13	GobDloopF: 5'-AAAGCATCGGTCTTGTAATC-3'	GobDloopR: 5'-CTTGGCTAGGCGTCTTGG -3'	53.0 ºC	(Chai et al., 2020)

Supplementary Table S3. Data sources of mitochondrial genomes used for time-calibrated Bayesian phylogeny.

Genus	Species	Collection locality	Genbank No.	References
Sarcocheilichthys	*Sarcocheilichthys parvus*	Yongjia County, Zhejiang Province, China	JX456224	(Chen et al., 2013)
	Sarcocheilichthys caobangensis	Chongzuo City, Guangxi Province, China	ON963980	This study
	Sarcocheilichthys sinensis		KJ850307	Unpublished
	Sarcocheilichthys nigripinnis	Huangshan City, Anhui Province, China	KJ997940	(Wang et al., 2016a)
	Sarcocheilichthys kiangsiensis		KY779851	Unpublished
	Sarcocheilichthys variegatus microoculus	Japan	AB054124	(Saitoh et al., 2003)
Hemibarbus	*Hemibarbus nummifer*	Wuyishan City, Fujian Province, China	KJ413052	(Wang et al., 2016b)
	Hemibarbus longirostris	Ganwon-do, South Korea	DQ347952	(Kim et al., 2009)
Squalidus	*Squalidus longifilis*	Fengcheng City, Liaoning Province, China	MT767747	(Chai et al., 2020)
Microphysogobio	*Microphysogobio yaluensis*	South Korea	KR075133	(Park et al., 2016)

	Microphysogobio rapidus	South Korea	MH713708	(Kim et al., 2020)
Biwia	*Biwia zezera*	Shiga, Moriyama, Japan	AB250108	(Horikawa et al., 2007)
Huigobio	*Huigobio chenhsienensis*	Ningdu County, Jiangxi Province, China	KU314694	(Li et al., 2018)
Abbottina	*Abbottina binhi*	Pingguo County, Guangxi Province, China	MK852688	(Chen et al., 2019)
Pseudogobio	*Pseudogobio anderssoni*	Anyi County, Jiangxi Province, China	MN883563	(Fu et al., 2020)
	Pseudogobio guilinensis	Pingle County, Guangxi Province, China	MN883564	(Fu et al., 2020)
Saurogobio	*Saurogobio dabryi*	Xiushui County, Jiangxi Province, China	KU314696	(Li et al., 2018)
	Saurogobio gymnocheilus	Hengdong County, Hunan Province, China	MK860911	(Tong et al., 2019)
Gobio	*Gobio acutipinnatus*	Bu'erjin, Xinjiang Uygur Autonomous Region, China	MT632635	(Yi et al., 2020)
	Gobio gobio	Plana, Czech Republic	AB239596	(Saitoh et al., 2006)
Mesogobio	*Mesogobio lachneri*	Linjiang City, Jilin Province, China	OL678457	(Tian et al., 2022)
Acanthogobio	*Acanthogobio guentheri*		MF787799	Unpublished
Romanogobio	*Romanogobio ciscaucasicus*		AP011259	Unpublished
Gnathopogon	*Gnathopogon elongatus*		AB218687	(Saitoh et al., 2006)
	Gnathopogon imberbis		KM086722	(Tao et al., 2016)
Coreoleuciscus	*Coreoleuciscus splendidus*	South Korea	EU848546	(Kim et al., 2012)
	*Coreoleuciscus aeruginos**	South Korea	DQ347951	(Kim et al., 2012)
Pseudorasbora	*Pseudorasbora elongata*	Wuyuan County, Jiangxi Province, China	KF245485	(Chen et al., 2015)
Pungtungia	*Pungtungia herzi*	South Korea	KF006339	(Yu et al., 2014)
Pseudopungtungia	*Pseudopungtungia nigra*	South Korea	EU332752	(Hwang et al., 2014a)
Paracanthobrama	*Paracanthobrama guichenoti*		KJ645748	(Du et al., 2016)
Gobiobotia	*Gobiobotia macrocephala*	South Korea	FJ515918	(Hwang et al., 2014b)
Xenophysogobio	*Xenophysogobio nudicorpa*	Jiangjin District, Chongqing City, China	KU314698	(Li et al., 2018)
Coreius	*Coreius guichenoti*	Banan District, Chongqing City, China	JF906108	(Xu et al., 2013)
	Coreius heterokon	Banan District, Chongqing City, China	JF906110	(Xu et al., 2013)
Ladislavia	*Ladislavia taczanowskii*	Kuandian County, Liaoning Province, China	MT897995	(An et al., 2021)
Acheilognathus	*Acheilognathus macropterus*	Wuhan City, Hubei Province, China, China	KJ499466	(Zhu et al., 2016)
Rhodeus	*Rhodeus sericeus*	Xiaoling'anling, Heilongjiang Province, China	KM052222	(Xu et al., 2016)
Tanakia	*Tanakia limbata*	Okayama, Japan	KM386633	(Luo et al., 2016)

* The scientific name in Genbank is *Coreoleuciscus splendidus*, and Song and Bang (2015) revised it as *Coreoleuciscus aeruginos*.

Supplementary Table S4. Model comparison for ancestral-area estimations of *Sarcocheilichthys parvus* using BioGeoBEARS. *d* = dispersal; *e* = extinction; *j* = founder event speciation. The optimal model is in a bold.

**Model**	**LnL**	**numparams**	***d***	***e***	***j***	**AIC**	**AIC_wt**
DEC	-78.57	2	0.16	1.00E-12	0.00E+00	161.1	4.10E-02
DEC+J	-121.5	3	0.14	3.50E-02	7.50E-03	248.9	3.60E-21
DIVALIKE	-75.42	2	0.20	1.00E-12	0.00E+00	154.8	9.60E-01
DIVALIKE+J	-126.8	3	0.16	1.00E-12	1.00E-02	259.6	1.70E-23
BAYAREALIKE	-158.3	2	0.32	1.33E+00	0.00E+00	320.5	1.00E-36
BAYAREALIKE+J	-132.6	3	0.13	1.60E-01	1.40E-02	271.3	5.00E-26

Reference

An, Q., and Fu, C. Z. (2021). Complete mitochondrial genome of *Ladislavia taczanowskii* (Cypriniformes: Gobionidae). *Mitochondrial DNA B* 6(8), 2227-2228. doi:10.1080/23802359.2021.1944385

Chai, J., and Fu, C. Z. (2020). Three mitochondrial genomes of freshwater fishes in the genus *Squalidus* (Cypriniformes: Gobionidae). *Mitochondrial DNA B* 5(3), 3779-3781. doi:10.1080/23802359.2019.1623728

Chen, A. H., Xia, R., Lei, G. C., Fu, C. Z. (2015). Complete mitochondrial genome of *Pseudorasbora elongata* (Cypriniformes: Cyprinidae). *Mitochondrial DNA* 26(2), 250-251. doi:10.3109/19401736.2013.823184

Chen, X., Zhou, Z. M., Chen, Z. J., & Ai, W. M. (2013). Complete mitochondrial genome of *Sarcocheilichthys parvus* (Cypriniformes, Cyprinidae). *Mitochondrial DNA* 24(2), 97-98. doi:10.3109/19401736.2012.723000

Chen, Y., and Fu, C. Z. (2019). Three complete mitochondrial genomes of freshwater fishes in the genus *Abbottina* (Cypriniformes: Gobionidae). *Mitochondrial DNA B* 4(2), 2179-2180. doi:10.1080/23802359.2019.1623728

Du, F. K., Xu, G. C., Nie, Z. J., Xu, P., & Gu, R. B. (2016). Complete mitochondrial genome of *Paracanthobrama guichenoti*. *Mitochondrial DNA A* 27(1), 727-728. doi:10.3109/19401736.2014.913161

Fu, J. W., and Fu, C. Z. (2020). Three mitochondrial genomes of *Pseudogobio* fishes (Cypriniformes: Gobionidae). *Mitochondrial DNA B* 5(3), 3064-3065. doi:10.1080/23802359.2020.1797553

Horikawa, M., Nakajima, J., Mukai, T. (2007). Distribution of indigenous and non-indigenous mtDNA haplotypes of *Biwia zezera* (Cyprinidae) in northern Kyushu, Japan. *Japanese Journal of Ichthyology* 54(2), 149-159.

Hwang, D. S., Byeon, H. K., Lee, J. S. (2014b). Complete mitochondrial genome of the freshwater gudgeon, *Gobiobotia macrocephala* (Cypriniformes; Cyprinidae). *Mitochondrial DNA* 25(1), 31-32. doi:10.3109/19401736.2013.775275

Hwang, D. S., Lee, W. O., Lee, J. S. (2014a). Complete mitochondrial genome of the freshwater gudgeon, *Pseudopungtungia nigra* (Cypriniformes, Gobioninae). *Mitochondrial DNA* 25(1), 1-2. doi:10.3109/19401736.2013.775260

Kim, K. R., and Bang, I. C. (2020). Complete mitochondrial genome of the endangered freshwater fish *Microphysogobio rapidus* (Cypriniformes, Cyprinidae) from Korea. *Mitochondrial DNA B* 5(1), 447-448. doi:10.1080/23802359.2019.1704642

Kim, K. Y., Lim, Y. H., Bang, I. C., Nam, Y. K. (2009). Phylogenetic relationships among three new *Hemibarbus* mitogenome sequences belonging to the subfamily Gobioninae (Teleostei, Cypriniformes, and Cyprinidae). *Mitochondrial DNA* 20(5-6), 119-125. doi:10.3109/19401730903176896

Kim, K. Y., Nam, Y. K., Bang, I. C. (2012). Phylogeny and divergence time estimation of *Coreoleuciscus splendidus* populations (Teleostei: Cypriniformes) endemic to Korea based on complete mitogenome sequences. *Genes. Genom.* 34(2), 149-156. doi:10.1007/s13258-011-0098-x

Li, Y. H., Cao, K., Fu, C. Z. (2018). Ten fish mitogenomes of the tribe Gobionini (Cypriniformes: Cyprinidae: Gobioninae). *Mitochondrial DNA B* 3(2), 802-803. doi:10.1080/23802359.2018.1467236

Luo, Y., Cao, X. J., Zhu, Y. R. (2016). The complete mitochondrial genome of *Tanakia limbata* (Cypriniformes: Cyprinidae). *Mitochondrial DNA A* 27(3), 1713-1714. doi:10.3109/19401736.2014.961135

Park, C. E., Park, G. S., Kim, M. C., Kim, K. H., Park, H. C., Lee, I. J., Shin, J. H. (2016). Complete mitochondrial genome of the Korean endemic species *Microphysogobio yaluensis* (Teleostei, Cypriniformes, Cyprinidae). *Mitochondrial DNA A* 27(5), 3557-3559. doi:10.3109/19401736.2015.1074217

Saitoh, K., Miya, M., Inoue, J. G., Ishiguro, N. B., Nishida, M. (2003). Mitochondrial genomics of ostariophysan fishes: Perspectives on phylogeny and biogeography. *J Mol. Evol.* 56(4), 464-472. doi:10.1007/s00239-002-2417-y

Saitoh, K., Sado, T., Mayden, R. L., Hanzawa, N., Nakamura, K., Nishida, M., Miya, M. (2006). Mitogenomic evolution and interrelationships of the Cypriniformes (Actinopterygii : Ostariophysi): The first evidence toward resolution of higher-level relationships of the world's largest freshwater fish clade based on 59 whole mitogenome sequences. *J. Mol. Evol.* 63(6), 826-841. doi:10.1007/s00239-005-0293-y

Song, H. Y., and Bang, I. C. (2015). *Coreoleuciscus aeruginos* (Teleostei: Cypriniformes: Cyprinidae), a new species from the Seomjin and Nakdong rivers, Korea. *Zootaxa 3931*(1), 140-150.

Tao, W. J., and Zhao, H. P. (2016). The complete mitogenome of *Gnathopogon polytaenia* (Cypriniformes; Cyprinidae). *Mitochondrial DNA A* 27(2), 1307-1308. doi:10.3109/19401736.2014.945569

Tian, W., Ni, X. M., Fu, C. Z. (2022). The complete mitochondrial genome of *Mesogobio lachneri* (Cypriniformes: Gobionidae) from Northeast Asia. *Mitochondrial DNA B* 7(10), 1810–1813. dio: 10.1080/23802359.2022.2131370

Tong, J., and Fu, C. Z. (2019). Four complete mitochondrial genomes of *Saurogobio* fishes (Cypriniformes: Gobionidae). *Mitochondrial DNA B* 4(2), 2175-2176. doi:10.1080/23802359.2019.1623126

Wang, F. Y., Jean, C. T., Chen, Y. J., Lin, K. Y., Liu, M. Y. (2016b). The complete mitochondrial genome sequence of *Belligobio nummifer* (Cypriniformes, Cyprinidae). *Mitochondrial DNA A 27*(1), 435-436. doi:10.3109/19401736.2014.898293

Wang, H., He, L. P., Yang, X. X., Li, C., Gu, J. W., Wang, X. R., et al. (2016a). Identification of the mitogenome of *Sarcocheilichthys nigripinnis* (Cypriniformes: Cyprinidae). *Mitochondrial DNA A* 27(2), 1225-1226. doi:10.3109/19401736.2014.945529

Xu, C. Z., Xie, F., Zhang, X., Zhao, S. (2016). The complete mitochondrial genome of Amur bitterling (*Rhodeus sericeus*) from China. *Mitochondrial DNA A 27*(4), 2377-2378. doi:10.3109/19401736.2015.1028038

Xu, D. D., Li, P., Zhang, Y. G., Peng, Z. G. (2013). Comparative study of the complete mitochondrial genomes of the bronze gudgeon (*Coreius heterodon*) and largemouth bronze gudgeon (*Coreius guichenoti*). *Mitochondrial DNA* 24(3), 189-190. doi:10.3109/19401736.2012.744979

Yang, X. S., Ni, X. M., Fu, C. Z. (2022). Phylogeographical Analysis of the Freshwater Gudgeon *Huigobio chenhsienensis* (Cypriniformes: Gobionidae) in Southern China. *Life-Basel 12*(7), 16. doi:10.3390/life12071024

Yi, T. Y., and Fu, C. Z. (2020). Two mitochondrial genomes of freshwater gudgeons in the genus *Gobio* (Cypriniformes: Gobionidae). *Mitochondrial DNA B* 5(3), 3072-3073. doi:10.1080/23802359.2020.1797569

Yu, J. N., Kim, S., Kwak, M. (2014). Complete mitochondrial genome sequence of a Korean *Pungtungia herzi* (Cypriniformes, Gobioninae). *Mitochondrial DNA* 25(6), 414-415. doi:10.3109/19401736.2013.809435

Zhu, X. P., Ma, Z. H., Yang, X. F., Xu, H. X., Yang, R. B. (2016). Complete mitochondrial genome of the Chinese bitterling *Acheilognathus macropterus* (Cypriniformes: cyprinidae). *Mitochondrial DNA A* 27(1), 589-590. doi:10.3109/19401736.2014.908358
